# Development of Cut Scores for Feigning Spectrum Behavior on the Orebro Musculoskeletal Pain Screening Questionnaire and the Perceived Stress Scale: A Simulation Study

**DOI:** 10.3390/jcm14155504

**Published:** 2025-08-05

**Authors:** John Edward McMahon, Ashley Craig, Ian Douglas Cameron

**Affiliations:** John Walsh Centre for Rehabilitation Research, Kolling Institute for Medical Research, School of Health Sciences, Faculty of Medicine and Health, The University of Sydney, St. Leonards, NSW 2065, Australia; a.craig@sydney.edu.au (A.C.); ian.cameron@sydney.edu.au (I.D.C.)

**Keywords:** orebro musculoskeletal pain screening questionnaire, perceived stress scale, simulation, malingering, feigning

## Abstract

**Background/Objectives**: Feigning spectrum behavior (FSB) is the exaggeration, fabrication, or false imputation of symptoms. It occurs in compensable injury with great cost to society by way of loss of productivity and excessive costs. The aim of this study is to identify feigning by developing cut scores on the long and short forms (SF) of the Orebro Musculoskeletal Pain Screening Questionnaire (OMPSQ and OMPSQ-SF) and the Perceived Stress Scale (PSS and PSS-4). **Methods**: As part of pre-screening for a support program, 40 injured workers who had been certified unfit for work for more than 2 weeks were screened once with the OMPSQ and PSS by telephone by a mental health professional. A control sample comprised of 40 non-injured community members were screened by a mental health professional on four occasions under different aliases, twice responding genuinely and twice simulating an injury. **Results**: Differences between the workplace injured people and the community sample were compared using ANCOVA with age and gender as covariates, and then receiver operator characteristics (ROCs) were calculated. The OMPSQ and OMPSQ-SF discriminated (ρ < 0.001) between all conditions. All measures discriminated between the simulation condition and workplace injured people (ρ < 0.001). Intraclass correlation demonstrated the PSS, PSS-4, OMPSQ, and OMPSQ-SF were reliable (ρ < 0.001). Area Under the Curve (AUC) was 0.750 for OMPSQ and 0.835 for OMPSQ-SF for work-injured versus simulators. **Conclusions**: The measures discriminated between injured and non-injured people and non-injured people instructed to simulate injury. Non-injured simulators produced similar scores when they had multiple exposures to the test materials, showing the uniformity of feigning spectrum behavior on these measures. The OMPSQ-SF has adequate discriminant validity and sensitivity to feigning spectrum behavior, making it optimal for telephone screening in clinical practice.

## 1. Introduction

Addressing psychosocial barriers to recovery plays a central role in the management of compensable injuries [1,2,3]. Chronic pain and associated syndromes, including opioid use disorder, represent a substantial cost to society, and these conditions are predicted to increase in the future [4]. The successful management of chronic pain, if possible, would result in significant economic savings, reduction of personal suffering, and an increase in productivity for the individual and society [4]. Similarly, feigning spectrum behavior (FSB) for the pursuit of secondary gain, such as work avoidance and access to pharmaceuticals, is detectable in 29% of personal injuries at formal psychological assessment [5]. As a medium of service delivery, telehealth has significant benefits in terms of convenience and capacity to reach clients in remote locations, bringing expertise otherwise unavailable due to distance. With the onset of Coronavirus, there was increased interest in telemedicine and adoption of these media for service delivery, rather than face-to-face consultation [6]. Yet little is known about how feigning spectrum behavior may manifest via telehealth [7,8]. Inherent to compensation systems is the notion of assigning a dollar value to injury, illness, and distress. In such a system the report of financial duress could be seen as a way of assessing the motivation or intent behind feigning spectrum behavior. Binder and Rohling conducted a meta-analysis showing a moderate effect size for financial incentives and reported abnormality and disability despite less severe injury in closed head injury [9]. Financial stress also plays a complex role in health. Structural equation modelling revealed that people reporting major financial stressors reported more interpersonal stress, psychological distress, and lower levels of psychological well-being [10]. This was associated with elevated interleukin 6, an inflammatory cytokine marker of stress [10]. In compensable injury there appears to be an inextricable relationship between the report of symptoms and the pursuit of secondary gains that may be exploited by some individuals.

The Orebro Musculoskeletal Pain Screening Questionnaire (OMPSQ) [11] is a widely used measure of the impact of injuries, and this scale indexes psychosocial factors that complicate recovery. It has moderate predictive power to identify persisting pain and disability in patients with back pain [2], sciatic leg pain [3], and acute and non-acute pain [12]. The Perceived Stress Scale (PSS) [13] has been correlated with a variety of factors, including health and smoking status and help-seeking behavior [14], and has been used to investigate work stress and associated health behaviors [15]. Both the OMPSQ [16,17,18,19,20] and PSS [21,22,23] have been validated in several languages, which is advantageous to the generalization of use in a multicultural society like Australia, although some qualitative research with culturally diverse people indicates that use of standardized questionnaires may have some unintended consequences, such as a negative impact on the client-practitioner relationship [24]. The multilingual validation of these measures demonstrates the robustness of the constructs measured by these scales and the impact of psychosocial factors on recovery and health regardless of culture and language. The OMPSQ and PSS have short forms, the ten-item OMPSQ-SF [25] and the four-item PSS-4 [14,26], respectively, which have similar predictive validity in health. The PSS has been validated for telephone use, and the OMPSQ-SF has been used telephonically; however, there has been no formal validation of the OMPSQ-SF for telephone administration. Unlike general health settings, the treatment of compensable injuries often involves complex interactions between the patient, treatment providers, agents of insurers, insurers, and legal service providers. Such complex systemic interactions can produce behaviors including malingering, factitious presentations, and false imputation of causes of conditions [27]. A comprehensive review of the literature across various search engines, including MEDLINE via OVID, Scopus, PsychInfo via OVID, Web of Science, and Google, produced no results for either the OMPSQ or PSS being validated with a simulation design to see if a feigned presentation can be distinguished from a genuine presentation.

The most successful method of detecting feigned mental disorder is the “Rare Symptoms” and “Quasi-rare symptoms” detection strategies [28]. These strategies detect feigning spectrum behavior by indexing the overreporting of infrequent symptoms or the reporting of symptoms to an exaggerated extent. That is, on measures of clinical symptoms, people engaging in feigning tend to endorse more pathology and rarer pathology than genuine respondents, producing inflated scores exceeding those generated by injured people reporting their pathology accurately. Usually, this strategy of detection generates a large to very large effect size [29]. Simulation studies involve the determination of cut-points for feigning based on the probability of genuine responding by comparing people with the condition to people without the condition who were asked to fake the condition. Criterion-referenced, or “known group” studies involve classifying people with the condition and those without the condition by some means, such as another measure or a known diagnosis, and then administering the subject measure. Research on the Minnesota Multiphasic Personality Inventory-Restructured Format (MMPI-RF) shows that both simulation studies and criterion-referenced studies generate large effect sizes [29,30]. Similar effect sizes were also found regardless of what disorder or symptom was to be faked. However, the effect size reduced to the moderate range if the simulator was educated about the disorder or the measure, so-called “sophisticated” simulators [30,31].

An alternative strategy to detecting feigning spectrum behavior is forced choice testing, and this has been applied to cognitive and memory impairments and malingered pain [32], using the Test of Memory Malingering (TOMM). However, the medium of telephone limits the stimuli to auditory, and the TOMM is a visual task. A verbal forced-choice test, akin to the 21-Item Test [33], a verbal list learning task, would offer a further detection strategy. This method of identifying feigning spectrum behavior was explored by the present study in a “simulation” design, and a 10-item Brief Forced Choice Test (BFCT) of verbal learning was created to test this method against the cut-score approach.

The present study aims to develop cut scores to identify FSB on the OMPQ and PSS and their short form and to see if a novel forced-choice strategy or financial rating can identify feigning. The design is a simulation study comparing the pattern of responding of injured workers (IW) with a community sample (CS) of non-injured people instructed to simulate a workplace injury (CSS) and respond genuinely (CSG). A further aim was to see if multiple exposures to test materials influenced the strategy to engage in FSB and if practice improved a non-injured person’s ability to simulate an injury. The study also investigated the degree of reported financial duress to see if simulators equate financial distress with the intent to feign an injury and if there is any discriminant value in enquiring about financial status. It was hypothesized that the CSG will score significantly lower than IWs on all measures and that CSS will score significantly higher on all symptom measures and significantly lower on the BFCT and financial rating than IW.

## 2. Materials and Methods

### 2.1. Study Design and Participants

Given the expected large effect size and comparisons to be made, a sample size of 40 participants in each group would have sufficient statistical power [34] with a power estimate of 0.98 [35]. The IWs were the first 40 referrals who consented to pre-screening for an independent support service for people who had suffered compensable workplace injuries provided by Navigator Group, Sydney, Australia. The pre-screening included assessing suitability for the program, current treatment, social support, relationship with employer and insurer, and then the administration of the questionnaires. The CS was a sample of convenience of 40 non-injured people, including employees from other business units and willing associates. The CS was recruited by a business-wide e-mail requesting volunteers. CS participants were excluded if injured. Both groups were onboarded to the service by the first author, and their permission to participate was acquired orally for all telephone calls to be recorded “for quality, training and research purposes”. The administration of the questionnaires was explained as part of pre-screening, and a convenient time was scheduled for administration of the questionnaires by an intern psychologist. Each CS participant was scheduled under an alias into the workflow of the intern psychologist and instructed on which calls to respond to genuinely and when to simulate. Each CS participant responded to the assessment protocol four times, responding twice in the simulated condition (CSS) and twice genuinely (CSG). The order of simulation and genuine responding was counterbalanced to see if there were practice effects. In the simulation condition the community sample participants were instructed to “fake an injury that occurred in the course of your work”.

### 2.2. Measures

The OMPSQ is a 25-item measure that is scored by summing the ratings of 21 items that are Likert-type scales, which have various anchoring statements at the endpoints, yielding a range of scores from 4 to 210 [11], with scores over 105 predicting poor outcomes [36]. The OMPSQ-SF is a subset of 10 items of the parent scale, with scores greater than 50 predicting unfavorable outcomes [25]. These measures were administered as a set of verbal rankings using the anchoring statements and scaled out of 10 to yield totals as in the pen and paper measure. The PSS is a 10-item scale in which statements are rated from “never” to “very often” and it was interpreted dimensionally using the printed norms [13]. The PSS-4 short form is a 4-item scale using items 4, 5, 7, and 8 of the parent scale, and it was interpreted dimensionally using the printed norms. The short forms were calculated from the subset of items from the long forms of these scales. Consistent with the OMPSQ items, participants were asked to rate their financial well-being from 0 to 10, anchored from 0 = “very poor” to 10 = “good”. The BFCT was a 10-item list learning task with a free recall trial, a distraction trial of counting backward from 20 to 0, and then the forced choice trial [Appendix A lists the target and foil words]. Short rhyming pairs of words were selected to see if this would induce feigned misidentification.

### 2.3. Data Collection

An intern psychologist with honors-level qualifications was trained in the telephonic administration of the measures, with the calls made within a period from November to December 2017, when the program was initiated. The dataset was then compiled and de-identified and provided to the researchers by the manager of the support program. The study was conducted in Sydney, Australia, with the data deidentified and conveyed to the researchers in December 2019 after ethics approval was received on 29 June 2019.

### 2.4. Statistical Analysis

Statistical analysis was performed using IBM SPSS Statistics Version 26 (64-bit Edition). Shapiro–Wilk tests and Levene’s tests were used to assess for normality. Differences between subsets of the community sample were investigated using the Mann–Whitney U-test or *T*-tests depending on normality. Differences between the CS and IWs were compared for age using an independent groups T-test and for gender using a Bayesian Independent Groups analysis. Given the predicted directional relationship and large effect size, the IW, CSG, and CSS were compared using one-way analysis of variance, or if a marked difference between demographic variables was identified, then analysis of covariance (ANCOVA) would be conducted [37]. Given that the CSS and CSG were assessed on two occasions, the test-retest reliability was assessed using a two-way, single-measure absolute intraclass correlation for each measure. With a 95% confidence interval and large expected intraclass correlation coefficient for the CSG and CSS, a sample size of 40 (n > 37) retained power (Power > 0.80) [38]. Receiver operator characteristics (ROCs) and area under the curve (AUC) were calculated for the OMPSQ, OMPSQ-SF, PSS, and RSS-4. The sensitivity (Sn), specificity (Sp), positive predictive power (PPP), and negative predictive power (NPP) were calculated for viable cut scores.

## 3. Results

### 3.1. Demographic Differences

IW suffered non-trivial injuries consisting of 15 upper limb injuries, 13 lower limb injuries, 3 shoulder injuries, 9 back injuries, two lung injuries, and one “internal organs other”; with three of the IWs coded with two injuries. Table 1 outlines the demographic data describing the IWs and the CS. An independent-measures t-test revealed that the IWs were significantly older than the CS (t = 2.253, Sig. (2-tailed) = 0.027). Bayesian Independent Group analysis of the gender ratios in the groups revealed the ratio of males to females was statistically significantly different (t = −6.637, Sig. (2-tailed) = 0.000) with more females than males in CS. Table 1 shows the differences between the IWs and CS.

The Shapiro–Wilks test for normality in scores comparing males to females showed non-normality in CSG on five of the eight conditions comparing each gender on the PSS, PSS-SF, OMPSQ, and OMPSQ-SF. A subsequent Mann–Whitney U-test showed no significant differences between genders and responses on these measures in the CSG. The Shapiro–Wilks test for normality comparing males to females in the CSS showed normality for each condition and measure, and t-tests showed no significant differences on any of the measures. The Shapiro–Wilks test of normality in scores comparing employed to not employed for the CSG showed non-normality in four of the eight conditions on PSS, PSS-4, OMPSQ, and OMPSQ-SF. A subsequent Mann–Whitney U-test showed no significant differences between employed and not employed status in CSG and their test scores. The Shapiro–Wilks test of normality in scores comparing employed to not employed in CSS showed non-normality in PSS-4 for not employed, but the other seven conditions had normality. The three t-tests and one Mann–Whitney U-test showed no significant differences between those employed and not employed within CSS and their test scores.

### 3.2. Analysis of Covariance Between Conditions

Table 2 contains the means for each condition and the results of the analysis of covariance with covariates of gender and age. The OMPSQ and OMPSQ-SF discriminated between the responses of IW, CSG, and CSS. However, the PSS, PSS-4, and finance question only discriminated between the CSS and the groups responding genuinely. Gender and age did not significantly contribute to scores on OMPSQ (sig. = 0.320/0.694), OMPSQ-SF (sig. = 0.637/0.637), PSS (sig. = 0.650/0.988), PSS-4 (sig. = 0.547/0.707), or Forced Choice Total (sig. = 0.115/0.444), but gender contributed to variance in the Finance Rating (sig. = 0.027).

### 3.3. Short-Term Test-Retest Reliability

The test-retest reliability for the CSG and CSS conditions was acceptable, revealing uniform behavior across the different conditions with telephone administration, and the results of this analysis are shown in Table 3. On the BCFT, the GSG had poor test-retest reliability (r = 0.115, *p* = 0.481, X = 9.2, SD = 1.02, range [5,6,7,8,9,10]), and the GSS had moderate reliability (r = 0.644, *p* < 0.001, X = 8.82, SD = 1.70, range [1,2,3,4,5,6,7,8,9]).

### 3.4. Receiver Operator Characteristics

**Figure 1:**
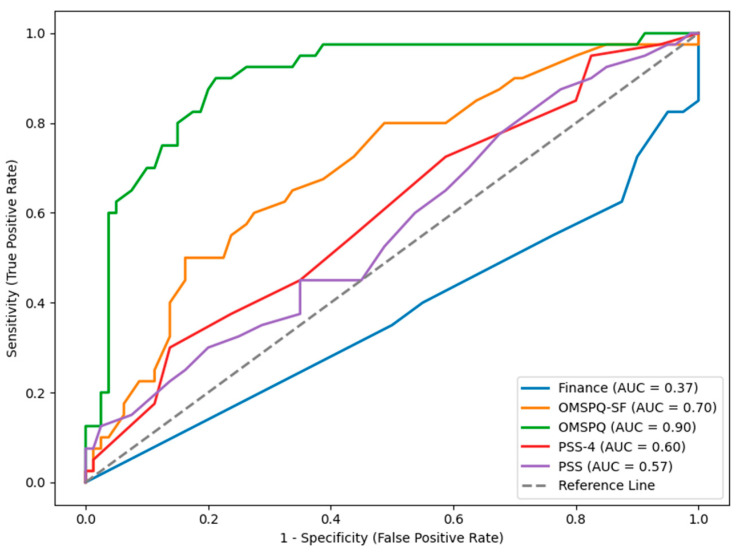
Receiver operator characteristics curve for injured workers (IW) versus community sample genuine (CSG).

**Figure 2:**
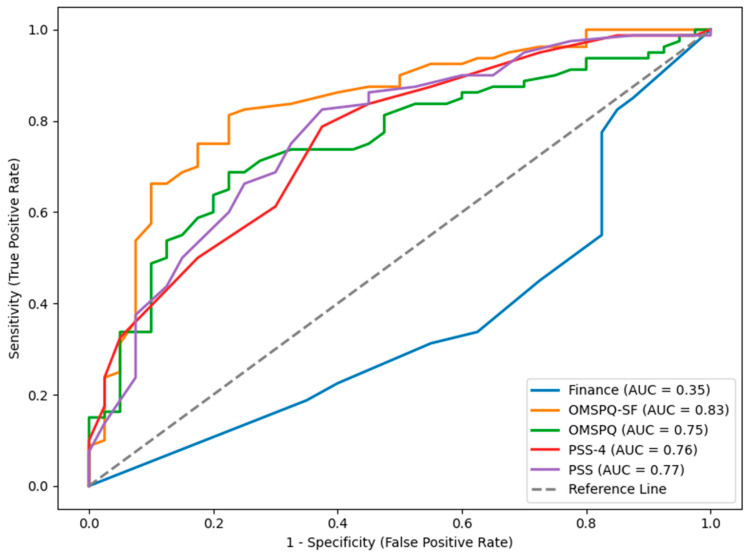
Receiver operator characteristics (ROCs) curve for injured workers (IW) versus community sample simulating (CSS).

“Elbow” points are where the plot line of the ROCs curve approaches 90 degrees, which indicates that the test score clearly delineates between the two samples. Visual inspection of the ROCs curves in Figure 1 shows that the OMPSQ has acceptable “elbow” points for identification of cut scores to differentiate between CSG and the IWs and that the OMPSQ SF has some opportunities but tends towards a more dimensional interpretation. The other scales show little opportunity for cut scores. Figure 2 shows the OMPSQ and OMPSQ-SF have shapes that indicate likely cut-points for distinguishing CSS from IW.

Table 4 contains the ROCs of the CSG and CSS compared with the IW. Comparing the lower bound of the confidence interval of the estimated AUC as an assessment of the overall model quality showed that the OMPSQ (0.83) and OMPSQ-SF (0.60) discriminated between the IWs and CSG, but the other measures were not better than chance at classifying membership to this group. The PSS (0.68), PSS-4 (0.67), OMPSQ (0.66), and OMPSQ-SF (0.76) classified CSS from IW, with the OMPSQ-SF being the best for this determination.

A cut score for distinguishing CSG and IWs on the OMPSQ of 15 had an Sn of 97.5% and an SP of 90%, with a PPP of 2.5% and an NPP of 100%. A cut score of 51 had 90% Sn, 23.8% Sp, a PPP of 92.5%, and an NPP of 78.75%, which shows the broad range of scores in the IWs sample. For discriminating between CSG and IW, the OMPSQ-SF at a cutoff at 20 had an Sn of 95%, 80% Sp, PPP of 97.5%, and NPP of 21.25%.

Table 5 and Table 6 contain the prospective cut scores for discriminating the IWs sample from the CSS with Sn, Sp, NPP, and PPP at each of the upper limits of the bands identified by Linton, Nicholas, and MacDonald [25] for the OMPSQ and OMPSQ-SF, respectively. Using a specificity of greater than 95% to minimize false positives, the OMPSQ using a cut score of 140 identified 28.8% of the CSS and 5% of the IWs sample as engaging in FSB. The OMPSQ-SF, using a cut score of 70, identified 25% of CSS with a 5% identification of IWs as engaging in FSB. Given base rates of FSB [39], it is probable that these are true positive identifications of feigning in this IWs sample.

## 4. Discussion

The present study compared methods for identifying feigning spectrum behavior from genuine responding on variants of the OMPSQ and PSS and other possible novel strategies, including a forced-choice auditory memory procedure and a question about finance delivered telephonically. The OMPSQ had sensitivity to discriminate between non-injured people, people with significant workplace injuries, and non-injured people asked to simulate an injury. The OMPSQ-SF appears to have a “Goldilocks” combination of questions that distinguishes between uninjured community sample members responding genuinely, workplace injured people, and the community sample when asked to simulate an injury with the minimum number of questions. While the PSS and PSS-4 statistically distinguished between the simulation conditions and people injured in the workplace, these measures did not have good ROCs for cut scores to identify feigning. Therefore, the PSS and PSS-4 scores should not be relied upon solely as indications of FSB but may contribute in combination with other measures. Similarly, conclusions about high scores on the PSS and PSS-4 cannot be concluded to simply reflect stress but may represent FSB, and furthermore, the constructs measured by these tests are falsifiable or able to be feigned without being distinguished from genuine responding.

Using the Linton et al. 2003 [11] and 2011 [25] cut scores of 105 and 90, respectively, 8 and 19 of IWs were classified at risk of poor adjustment on the OMPSQ, and 51/80 and 62/80 of the CSS condition were classified as at risk for poor adjustment. The OMPSQ-SF cut-off of 50 classified 7 of the IWs and 60/80 of the CSS as at risk for poor adjustment. This shows the differing sensitivities of these cut scores derived from the OMPSQ, with the OMPSQ-SF best resembling the original cut score sensitivity for poor adjustment. Scores above 70 on the OMPSQ-SF, identifying less than 5% of IW, likely reflect feigning spectrum behavior, and 20 of the CSS would have been identified as simulating and 2 of the IW.

Examining the different demographic variables of the CS statistically showed no differences between variables of age and employment status on the pattern of responding in CSG and CSS conditions. Given the counterbalanced order of administration of test conditions to the CS, the strong intraclass correlation, and that there were no significant differences between Trial 1 or Trial 2 for CSS, this suggests people tend to engage in the same kind of feigning spectrum behavior regardless of previous exposures to the test material. The data also suggests the OMPSQ and the OMPSQ-SF appear to retain sensitivity to feigning despite repeated administration and exposure to the test materials and the opportunity to practice simulation. Other longer measures, such as the MMPI-2, have reduced sensitivity to identify feigning spectrum behavior with coaching or repeated administrations [29,30]. The CSG inherently endorsed financial duress when simulating, and more so after previous exposure to the telephone protocol. This shows, within simulated compensation scenarios, people naturally equate distress with financial duress despite there being no inherent suggestion of such a relationship. Enquiring about financial well-being using a 10-point Likert Scale has some utility in identifying attitude to recovery in compensable samples, with scores below 5 a possible negative indicator. The CSS did not identify memory loss as a symptom to feign when asked to simulate a workplace injury. This may have been because the task seemed too easy, that the task seemed unrelated to the simulation of a work injury, or that it was easily identified as a possible detection strategy. Alternately, the forced choice task may have failed because the 10 items were not enough items to create the illusion that an injured person would reasonably perform poorly on it, so CSS did not try and fail this measure. At 50 items, the TOMM realistically creates the illusion of being fallible by being beyond free recall and requiring recognition, and at 10 items, the BFCT may not have created such a cognitive illusion.

The goodness of the ROCs for identifying CSG on the OMPSQ and OMPSQ-SF was generally better than for the prediction of long-term recovery, with the AUC in studies of recovery ranging from 0.586 to 0.710 [7,23]. This likely reflects the increased accuracy of identifying a state rather than a trait in construct measurement, and it is possible that at times of measurement, these studies were confounded by low “trust” states resulting in feigning spectrum behavior. The compensation status of the participants in these other studies was not identified, and it is possible the presence of compensation can distort the predictive accuracy. For example, applying the cut scores identified above to Linton et al. [23] published results would identify 19% of those who had more than 14 days of accumulated sick leave and 2.7% of those who did not have time off as engaging in feigning spectrum behavior.

## 5. Limitations

Limitations exist in the current study. A confound of the study was that there was only one telephone assessor, and the community sample were likely recognizable by voice such that a true blind could not be maintained across the experiment. A further limitation was the statistically significant gender and age differences between the IWs and CS. This reflects volunteer effects and limitations to this kind of research. Although this did not generate statistically significant differences in responding. The ratio of males to females in the CS was roughly equivalent to the Primary Care Sample in the development of the OMPSQ-SF [25]. Research on gender and feigning spectrum behavior is somewhat limited; however, recent research in both clinical and forensic settings found males tended to engage in malingering more than females [40,41], and of those deemed to be engaging in malingering as detected by an interview using the “rare” and “quasi rare” method, the Miller-Forensic Assessment of Symptoms Test, males and females did not differ significantly on their pattern of feigning behavior, except that female’s reports of hallucinations were more likely to be considered genuine rather than male’s by expert examiners [40]. In a litigating Australasian sample, the Infrequency (F) scale on the MMPI-2 was used to compare the base rate of malingering in females and males [39], and despite more males comprising the normative sample (57%), females were as likely to be identified as feigning as males. In the present study ANCOVA showed no significant effect for gender or age on the classification of simulating. The CS was significantly younger than the work-injured sample; however, statistically, age did not significantly contribute to variance. The research on age and feigning spectrum behavior indicates that young adults are more likely to admit to engaging in feigning spectrum behavior [42], and future research using the cut scores identified above could test “empathic confrontation” type interventions with young adults breaching the cut scores. Some research suggests that age matching and race matching of simulated malingerers to actual malingering populations are not necessary because the differences are not large enough to influence clinical interpretation of the pattern of responding [43], although performance validity measures doing double duty to detect malingering are improved with age-corrected scores [44]. On inspection, there were educational differences between the IWs and CS. Research on FBS shows this behavior is detected more often in people with lower levels of education [45], and sophistication, which may be assumed from more educated people, reduced the effect size of simulation studies [30]. The effects of education status on ability to engage in FBS warrant further examination by well-defined studies, and it is possible that this factor reduced the sensitivity of the cut scores identified. A further limitation of the current study was the same as in the development of the OMPSQ Short Form; that is, the short forms and statistics were calculated from the administration of the long form, and it is not known how completing only the short form version alone might affect responses, and repeating the study with the short forms alone would contribute to the validity of their use.

## 6. Conclusions

This study adds to the nascent body of research on the use of telephone-administered psychometric tests to detect FSB and contributes to the literature on the OMPSQ, OMPSQ-SF, PSS, and PSS-4. Future research with larger sample sizes could examine the relative utility of the cut scores developed above with more diverse samples. Given the utility and general acceptance of the OMPSQ by workers compensation systems, further research into the characteristics of simulated injury is warranted, with the present study representing an initiation of this process. Similar research would benefit from the use of debriefing interviews to inform on the simulator’s strategies and sensitivity to the questions and methods. Similarly, empathic confrontation and debriefing of injured workers identified as engaging in feigning spectrum behavior would inform about the psychology and approaches of high-stakes malingerers within compensation systems. Future research, using these or other cut-offs for feigning spectrum behavior, could tailor interventions to address the motivational factors underlying the breaching of such cut scores. By addressing FSB early in recovery and assisting forensic examiners in retrospective evaluation of these factors in recovery, these and similar cut scores can reduce unnecessary costs to society often left unchecked in our compensation systems.

## Figures and Tables

**Table 1:** Demographic differences between injured workers (IW) and community sample (CS).

Variable	Injured Workers	Community Sample
N	40	40
Age (SD, Median, IQR, Max and Min)	41.575 (12.226, 39, 18, Min = 18 Max = 66)	34.95 (14.016, 28, 24, Min = 19 Max = 72) *
Gender: Male/Female	33 (82.5%)/7 (17.5%)	9 (22.5%)/31 (77.75%) *
Professional with Degree	5 (12.5%)	14 (25%)
Qualified Trades (mechanics, chefs, etc.)	14 (35%)	11 (27.5%)
General Labor/Farm Worker/Machine Operator/Driver	14 (35%)	0 (0%)
Mining Industry	5(12.5%)	0 (0%)
Hospitality	3(5%)	2 (5%)
Students		8 (20%)
Mean days from injury to	38.7 (13.2)	N/A

* Significant differences (Sig. >0.05).

**Table 2:** Means, standard deviations, ranges of scores and analysis of covariances for age and gender.

Test Score	GSG Trial 1 Mean (SD) [Range]	CSG Trial 2 Mean (SD) [Range]	IWMean (SD) [Range]	CSS Trial 1 Mean (SD) [Range]	CSS Trial 2 Mean (SD) [Range]
OMPSQ	38.67 *^CDE^ (21.87) [7–113]	40.50 *^CDE^ (22.89) [4–112]	84.15 *^ABCD^ (28.83) [14–152]	118.08 *^ABC^ (36.01) [46–192]	112.25 *^ABC^ (35.15) [32–186]
OMPSQ-Short Form	20.96 *^CDE^ (12.97) [1–63]	21.88 *^CDE^ (13.05) [2–58]	41.20 *^ABDE^ (13.87) [10–87]	59.73 *^ABC^ (18.93) [18–91]	56.18 *^ABC^ (18.32) [28–85]
PSS Total	13.11 *^DE^ (6.251) [1–23]	12.51 ^DE^ (6.76) [2–27]	14.94 *^DE^ (7.23) [2–30]	22.10 *^ABC^ (8.01) [0–39]	22.16 *^ABC^ (6.28) [8–36]
PSS-4 Total	4.55 *^DE^ (2.60) [0–9]	4.35 *^DE^ (2.78) [0–11]	5.45 *^DE^ (2.82) [0–12]	8.53 *^ABC^ (3.41) [0–15]	8.38 *^ABC^ (2.71) [3–15]
Finance Rating	8.43 *^DE^ (2.03) [2–10]	8.53 *^DE^ (1.96) [2–10]	6.85 *^DE^ (3.45) [0–10]	5.48 *^ABC^ (3.57) [0–10]	4.78 *^ABC^ (3.29) [0–10]
Forced Choice Test	9.35 (0.80) [7–10]	9.05 (1.20) [5–10]	9.30 (1.07) [6–10]	9.00 (1.78) [1–10]	8.65 *^AC^ (1.59) [3–10]

* Significance at 0.05 level, ^A^ = community sample genuine (CSG) Trial 1, ^B^ = CSG Trial 2, ^C^ = injured workers (IW), ^D^ = community sample simulating (CSS) Trial 1, ^E^ = CSS Trial 2.

**Table 3:** Short-term test-retest reliabilities for the OMPSQ, OMPSQ-SF, PSS, and PSS-4 of the community sample in two conditions administered twice.

Measure	ICC (95% CI)	*p*-Value	Minimal Detectable Change
OMPSQ	0.954 (0.943–0.964)	<0.001	21.719
OMPSQ-SF	0.905 (0.882–0.926)	<0.001	10.56
PSS	0.926 (0.908–0.942	<0.001	13.509
PSS-4	0.836 (0.791–0.874)	<0.001	6.114

**Table 4:** Receiver operator characteristics (ROCs) comparing community sample genuine (CSG) and community sample simulation (CSS) with injured workers (IWs).

Measure	Comparison	Area Under Curve	Std. Error	Asymptotic Significance	95% Confidence Interval Lower-Upper
OMPSQ	CSG v IW CSS v IW	0.897 0.751	0.032 0.461	<0.000 <0.001	0.834–0.959 0.661–0.849
OMPSQ-SF	CSG v IW CSS v IW	0.700 0.835	0.51 0.040	<0.001 <0.001	0.834–0.959 0.757–0.913
PSS	CSG v IW CSS v IW	0.567 0.771	0.056 0.046	0.235 <0.001	0.457–0.676 0.681–0.862
PSS-4	CSG v IW CSS v IW	0.587 0.760	0.055 0.046	0.078 <0.001	0.489–0.704 0.670–0.849
Finance	CSG v IW CSS v IW	0.370 0.354	0.055 0.540	0.180 0.007	0.263–0.478 0.247–0.460
BCFT	CSG v IW CSS v IW	0.545 0.581	0.056 0.056	0.425 0.150	0.434–0.656 0.471–0.691

**Table 5:** Cut scores, sensitivity (Sn), specificity (Sp), negative predictive power (NPP), and positive predictive power (PPP) from OMPSQ.

OMPSQ Cut Score [Range]	Sn ×100	1-Sp ×100	NPP %	PPP %
49 [0–50]	95.0	92.5	33	92.68
59 [51–60]	93.8	82.5	36.84	86.21
69 [61–70]	87.5	70.0	36.11	75.26
79 [71–80]	83.8	50.5	36.73	68.36
90 [81–90]	75.0	45.0	33.87	59.41
99 [91–100]	68.8	25.0	35.36	48.18
110 [101–110]	53.8	12.5	32.38	37.72
120 [111–120]	47.5	10.0	31.85	33.04
126 [121–130]	37.5	10.0	29.75	26.09
140 [131–140]	28.8	5.0	28.57	18.80
150 [141–150]	16.3	2.5	26.89	10.17

**Table 6:** Cut scores, sensitivity (Sn), specificity (Sp), negative predictive power, and positive predictive power from OMPSQ.

OMPSQ-SF Cut Score [Range]	Sn ×100	1-Sp ×100	NPP %	PPP %
10 [0–10]	100	100.0	100	100
20 [11–20]	100	95.0	50	98.76
30 [21-30]	98.8	80.0	47.05	89.655
39 [31–40]	90.0	50.0	41.67	71.71
50 [41–50]	75.0	17.5	38.37	52.67
58 [51–60]	52.5	7.5	33.63	36.21
70 [61–70]	25.0	5	28.27	17.09
79 [71–80]	8.8	2.5	5.04	5.04

## Data Availability

The data presented in this study are available on request from the corresponding author. As the data was collected by Navigator Group Pty Ltd as part of business, it reserves the right to determine access to the data and the intent of the use of the data.

## Abbreviations

The following abbreviations are used in this manuscript:

FSB	Feigning spectrum behavior
SF	Short form
OMPSQ	Orebro musculoskeletal pain screening questionnaire
PSS	Perceived stress scale
PSS-4	Perceived stress sale–4 item
ANCOVA	Analysis of covariance
AUC	Area under the curve
ROC	Receiver operator characteristics
MMPI	Minnesota multiphasic personality inventory
RF	Restructured format
TOMM	Test of memory malingering
IW	Injured workers
CS	Community sample
CSS	Community sample simulating
CSG	Community sample genuine responding
BFCT	Brief forced choice test
Sn	Sensitivity
Sp	Specificity
PPP	Positive predictive power
NPP	Negative predictive power
SD	Standard deviation
Min	Minimum
Max	Maximum
N/A	Not applicable
Sig.	Significance

## Appendix A

**Table A1:** Target and foil words for brief forced choice test.

**Targe Words**	**Foil**
Cat	Rat
Boat	Coat
Rope	Hope
Van	Can
Book	Look
Hotel	Motel
Moose	Goose
Boot	Shoot
Pig	Jig
Tango	Mango
